# Common Mycorrhizal Network Induced JA/ET Genes Expression in Healthy Potato Plants Connected to Potato Plants Infected by *Phytophthora infestans*

**DOI:** 10.3389/fpls.2020.00602

**Published:** 2020-05-25

**Authors:** Pierre-Louis Alaux, Françoise Naveau, Stéphane Declerck, Sylvie Cranenbrouck

**Affiliations:** ^1^Earth and Life Institute, Applied Microbiology, Mycology, Université catholique de Louvain, Louvain-la-Neuve, Belgium; ^2^Earth and Life Institute, Applied Microbiology, Mycology, Mycothèque de l’Université catholique de Louvain, Université catholique de Louvain, Louvain-la-Neuve, Belgium

**Keywords:** common mycorrhizal network, arbuscular mycorrhizal fungi, *Phytophthora infestans*, Jasmonic acid pathway, potato

## Abstract

Most plants are connected belowground via common mycorrhizal networks (CMNs). In their presence, the transmission of warning signals from diseased to uninfected plants has been reported. However, current studies have all been conducted in pots making it difficult to discriminate direct from indirect contribution of hyphae to the transmission of the signals. Here, we conducted an *in vitro* study with potato plantlets connected by a CMN of the arbuscular mycorrhizal fungus *Rhizophagus irregularis*. The plantlets were grown in physically separated compartments and their connection ensured only by the CMN. The donor potato plantlets were infected by *Phytophthora infestans* and defense genes analyzed 24, 48 and 120 h post-infection (hpi) in the uninfected receiver potato plantlets. Twenty-four hpi by the pathogen, *PAL*, *PR-1b*, *ERF3*, and *LOX* genes were significantly upregulated, whereas no significant transcript variation was noticed 48 and 120 hpi. The exact nature of the warning signals remains unknown but was not associated to microorganisms other than the AMF or to diffusion mechanisms through the growth medium or induced by volatile compounds. The defense response appeared to be transitory and associated with the jasmonic acid or ethylene pathway. These findings demonstrate the direct involvement of hyphae in the transmission of warning signals from diseased to uninfected potato plantlets and their indubitable role in providing a route for activating defense responses in uninfected plants.

## Introduction

Plant roots establish complex interactions with microbial communities (Le Fevre and Schornack, 2016). Whereas soil bacterial and fungal communities interact with plants in many different ways (e.g., promoting or impacting plant productivity and health), the roles of arbuscular mycorrhizal fungi (AMF) are mostly associated with the improvement of plant nutrition (especially phosphorus) and alleviation of abiotic and biotic stresses (Pozo and Azcón-Aguilar, 2007; Pozo et al., 2010; Plouznikoff et al., 2016). The concept that these fungi may form common mycorrhizal networks (CMNs) interconnecting plants is nowadays widely recognized (Simard et al., 1997; Rhodes, 2017; Simard, 2018) suggesting their potential roles in nutrients exchange. Indeed, for ectomycorrhizal fungi, these belowground interconnections have been reported to favor nitrogen, phosphorus (He et al., 2003; Selosse et al., 2006), and carbon (Selosse and Roy, 2009) exchanges. However for AMF, the roles of CMNs in nutrients (e.g., phosphorus or nitrogen) or carbon transport among plants remain a matter of debate (Voets et al., 2008; Bücking et al., 2016).

In the recent decade, CMNs formed by AMF have been reported to improve plant defense by acting as channels for warning signals transmitted from plants impacted by pests or diseases to healthy neighbors (Johnson and Gilbert, 2015). Indeed, the production of volatile repellent compounds or elicitation of defense genes have been observed in healthy plants connected to plants attacked by insect herbivores (Babikova et al., 2013a; Song et al., 2014) or foliar necrotrophic fungi (Song et al., 2010). However, the nature of the signals conveyed by the CMNs remains speculative. Bacterial biofilms or microbial community shift (Barto et al., 2012; Babikova et al., 2013a) as well as chemicals or electric signals (Johnson and Gilbert, 2015) have been suggested, while no evidences of a role played by volatile organic compounds (VOCs) or root exudates (Song et al., 2010; Babikova et al., 2013a) have been reported.

Current studies on CMNs have all been conducted in pots with plants physically separated by 15/20 cm. The maximum response in the neighbor uninfected plants occurred usually between 48 and 100 h following attack or infection of the donor plant (Gilbert and Johnson, 2017) but first defense responses were recorded 18 h (Song et al., 2010) and even 6 h (Song et al., 2014) post infection by the pathogen. It is obvious that the benefit to the uninfected plants will be higher if the signal transmission is fast, enabling them to set up defense mechanisms prior to attack (Babikova et al., 2013b).

Pot culture systems have obvious limitations, namely, the possible presence of undesirable microbes that may affect the infected plant or uninfected neighbor plant or even the CMN. Diffusion of molecules and/or volatile compounds through the soil solution (e.g., not via the exclusive mycelium connecting the plants) is also a factor that could impact the response of the uninfected neighbor plant (Gilbert and Johnson, 2017). Therefore, pot culture systems could make it difficult to discriminate the direct contribution of hyphae to the transmission of warning signals from the indirect effects related to external factors (e.g., changes in microbial communities or transfer of chemical or volatile signals through the substrate). The utilization of contaminant-free *in vitro* cultivation systems with CMNs connecting plants separated by a physical wall may overcome part of these problems. Indeed, *in vitro* cultivation systems, although artificial, offer several advantages which are mainly, the absence of unwanted belowground contaminants potentially impacting the CMN or signal transfer and the strict physical separation between the donor and received compartments only connected by the hyphae crossing a partition wall, thus excluding transmission via diffusion of chemical or volatile compounds through the medium. Using *in vitro* cultivation systems may thus discriminate direct from indirect effects of the CMN on the transmission of warning signals.

In the last decade, *in vitro* cultivation systems have been used to investigate the interplant transport of carbon (Voets et al., 2008) or cesium (Gyuricza et al., 2010) between *Medicago truncatula* plantlets connected by CMNs. Interestingly, Gallou et al. (2011), used the Mycelium Donor Plant (MDP) *in vitro* culture system, following the name given by Voets et al. (2009), for the fast and homogenous colonization of potato plantlets and for studying their resistance against late blight caused by *Phytophthora infestans*. This study demonstrated the elicitation of defense genes in infected potato plantlets connected to potato donor plantlets free of the pathogen. Priming of *PR-1* and *PR-2* genes was observed in the receiver potato plantlets starting 48 h post inoculation (hpi) (Gallou et al., 2011) and a reduction of leaf infection and area under disease progress curves (AUDPC) were observed starting 24 hpi. Both observations clearly demonstrated that *Rhizophagus irregularis* MUCL 41833 triggered a systemic resistance in the leaves of potato plantlets, especially, during the first stage of infection. Similar results were obtained with soybean root pathogens (Marquez et al., 2018a, b). However, none of these studies considered the defense response in uninfected receiver plantlets connected to infected plantlets.

*Phytophthora infestans*, the causal agent of late blight, is an hemibiotroph pathogen known to produce several effectors during its biotrophic phase of infection, first suppressing plant defense responses and later inducing larger necrosis (Lee and Rose, 2010). In the present study, a CMN formed by the AMF *R. irregularis* MUCL 41833 was established *in vitro* between two potato plantlets, one donor infected by *P. infestans* and one receiver uninfected by the pathogen. We investigated whether the signal transmission was directly associated to the connecting hyphae and postulated that if the fungus can transmit signals from an infected to an uninfected potato plantlet, the latter will set up defense mechanisms likely similar to that of a direct infection by the pathogen. Therefore, the experiment was designed to study plant defense genes expression in an uninfected receiver potato plantlet connected by a CMN to an infected potato donor plantlet.

## Materials and Methods

### Biological Material

*In vitro* produced plantlets of *Solanum tuberosum* L. var. Bintje were provided by the “Station de Haute Belgique” (Libramont, Belgium). The plantlets were micropropagated every 4 weeks in culture microboxes, sealed with breathing filters in the lid (ref: 0118/120 + OD118, Combines, Belgium). The growth medium was Murashige and Skoog (MS) (Duchefa, Netherlands) supplemented with 20 g l^–1^ sucrose and solidified with 4.2 g l^–1^ Gelrite^TM^. The microboxes were placed in a growth chamber (Snijders Scientific B.V., Netherlands), under a relative humidity (RH) of 75%, a photoperiod of 16 h/8 h (day/night), a photosynthetic photon flux (PPF) of 24.5 μmol s^–1^ m^–2^, and a temperature of 20/18°C (day/night).

An *in vitro* culture of the AMF *R. irregularis* (Błaszk., Wubet, Renker, and Buscot) (Schüßler and Walker, 2010) as [“irregulare”] MUCL 41833 was provided by the Glomeromycota *in vitro* collection (GINCO^1^) on the Modified Strullu-Romand (MSR) medium (Declerck et al., 1998). The fungus was maintained on plantlets of *M. truncatula* Gaertn. cv. Jemalong A 17 (SARDI, Australia) in the MDP *in vitro* culture system (for details see Voets et al., 2009) until use.

An *in vitro* culture of the Oomycete *P. infestans* (mont.) de Bary MUCL 54981—mating type A1—was provided by the Mycothèque de l’Université catholique de Louvain (BCCM/MUCL^2^). It was initially isolated in July 2012 by the Centre Wallon de Recherches Agronomiques (CRAW, Libramont, Belgium) from a potato field in Temploux (Belgium). The strain was sub-cultured every 2 weeks in Petri plates (90 mm diam.) containing 20 ml of potato dextrose agar (PDA, Oxoid: CM0139) medium. Leaves from *in vitro* micropropagated potato plantlets (see above) were placed on the surface of actively growing colonies of *P. infestans* to stimulate the production of sporangia. The Petri plates were sealed with cellophane, wrapped in aluminum foil, and placed in the dark in a culture chamber set at 20°C 10 days before use.

### Experimental Design/Set Up

Potato plantlets were grown in the MDP *in vitro* culture system for root colonization by the AMF (see for details Voets et al., 2009). After 12 days, the plantlets were heavily colonized and transferred in the root compartment (RC) of bi-compartmented Petri plates (90 mm diam.) filled with 23 ml MSR medium without sucrose and vitamins and solidified with 3.2 g l^–1^ Gelrite. The AMF hyphae started to grow profusely out of the roots of the donor plantlets (named the mycorrhizal donor—D^+M^—plantlets) colonizing the RC. Medium was added to the RC at regular intervals to provide the plants with nutrients and to maintain the medium at the level of the top of the partition wall, facilitating hyphae to cross from the RC to the HC. Roots that passed the partition wall were trimmed. The hyphae started to cross the partition wall separating the RC from a hyphal compartment (HC—containing 25 ml MSR medium—just below the top of the partition wall) within 3 weeks. Fresh medium was added weekly to keep medium at the top of the partition wall in the RC. The growth front of the mycelium in the HC was homogenized by removing the MSR medium 2 cm away from the plastic wall and replacing it with fresh MSR medium (10 ml). After 1 week (i.e., 7 weeks of growth in the MDP *in vitro* culture system), 20 days old potato plantlets (i.e., named mycorrhizal receiver—R^+M^—plantlets) were placed in the HC with roots in contact with the mycelium growing front and shoot extending outside the Petri plate via hole. The plantlets were rapidly colonized by the AMF and common mycelium network linking plantlets from both compartments was established within a few days [i.e., the (D R)^+M^ treatment]. Control Petri plates [i.e., without AMF, the (D R)^–M^ treatment] were prepared strictly as above. The systems were placed in a growth chamber set at 75% RH, a photoperiod of 16 h/8 h (day/night), a PPF of 24.5 μmol s^–1^ m^–2^, and a °T of 20/18°C (day/night). Forty-eight MDP *in vitro* culture were considered for the (D R)^+M^ and (D R)^–M^ treatments.

**FIGURE 1:**
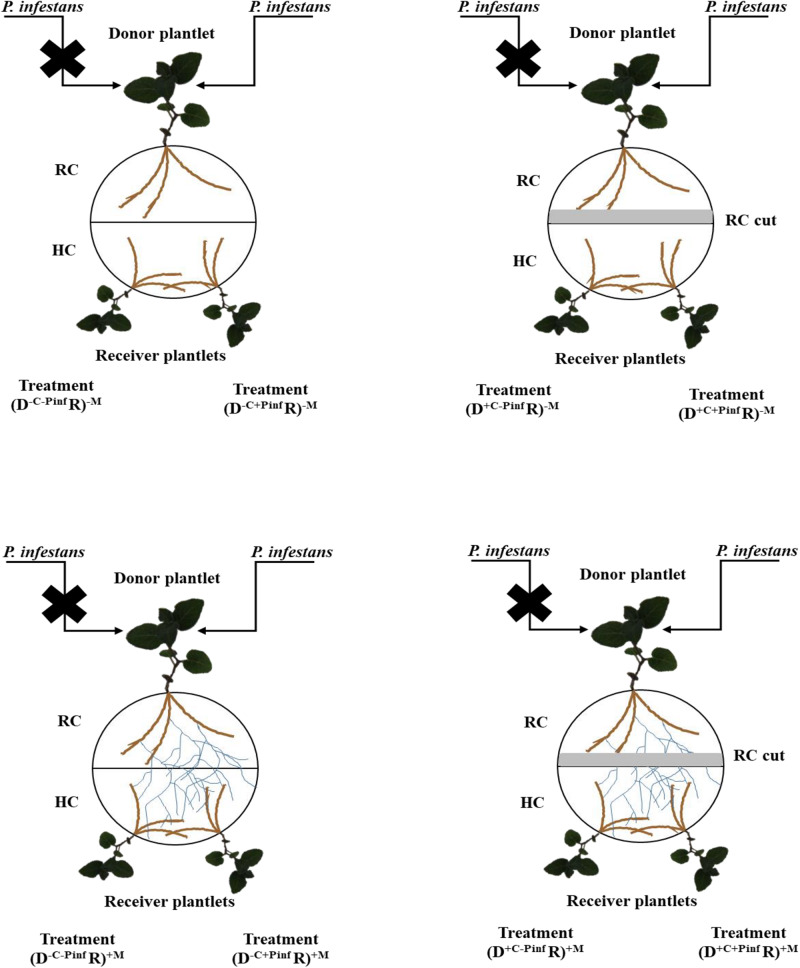
Schematic representation of the *in vitro* experimental system. Warning signal transmission was investigated from a donor (D) potato plantlet infected by *Phytophthora infestans* MUCL 54981 to two uninfected receiver (R) potato plantlets linked by a common mycorrhizal network of the arbuscular mycorrhizal fungus, *Rhizophagus irregularis* MUCL 41833. The plantlets developed in separate compartments (a root compartment, RC and a hyphal compartment, HC) physically separated by a plastic wall, while the AMF crossed the wall and interconnected the plants. Several treatments were considered, depending on whether the roots of the donor (D) plantlet were colonized or not by the AMF (+M, −M), the shoot infected of not by *P. infestans* (+Pinf, −Pinf), and the medium in the RC cut or not (+C, −C): (D^–C–Pinf^ R)^+M^, (D^+C–Pinf^ R)^+M^, (D^+C+Pinf^ R)^+M^ and (D^–C+Pinf^ R)^+M^, (D^+C–Pinf^ R)^–M^, (D^+C+Pinf^ R)^–M^, (D^–C+Pinf^ R)^–M^, (D^–C–Pinf^ R)^–M^.

After 8 days, the plantlets in the (D R)^+M^ treatment were randomly separated in two groups. In the first group, a piece of MSR medium of 0.5 cm width and 9 cm long (corresponding to the diameter of the Petri dish) was removed (i.e., cut – + C) along the partition wall of the RC, cutting CMN from the HC [i.e., (D^+C^ R)^+M^ treatment]. In the second group, no medium piece was cut (i.e., non-cut – −C) along the RC [i.e., (D^–C^ R)^+M^ treatment]. After an additional 4 days, half of the donor potato plantlets in each group was inoculated (+ Pinf) or not (−Pinf) with *P. infestans.* Inoculation was done with sporangia of *P. infestans* collected from 10 days old detached leaves of *in vitro* produced potato plantlets. Briefly, detached leaves from *in vitro* produced potato plants were placed in Petri plates on the surface of a culture of *P. infestans*. After 8 days, the leaves were removed and delicately cleaned with deionized sterilized (121°C for 15 min) water to harvest sporangia produced at their surface. Concentration of sporangia was adjusted to 100 units per μl^–1^. Sterilized cellophane disks of 0.5 mm diameter were placed on the adaxial part of the five oldest leaves of each donor potato plantlet, preventing evaporation and drop fall. Then, 20 μl of the sporangia solution was disposed between the cellophane and the leaf with a micropipette (Sartorius Stedim Biotec, France). Four treatments were thus considered (Figure 1), (D^+C+Pinf^ R)^+M^, (D^–C+Pinf^ R)^+M^, (D^+C–Pinf^ R)^+M^, (D^–C–Pinf^ R)^+M^. Each treatment was finally separated randomly in three groups of four replicates corresponding to three-harvesting times: 24, 48, and 120 hpi by *P. infestans*, which corresponded, to sporangia germination, first hyphae branching (initiation of tissue invasion) and apparition of the first necrotic lesions, respectively (Supplementary Figure S1). Twenty-four, 48, or 120 hpi by the pathogen, the leaves were removed from each receiver plantlet and transferred within 3 min to liquid nitrogen. The non-mycorrhizal potato plantlets were prepared strictly as above, and four treatments with four replicates per each harvesting time were identically set up (Figure 1): (D^–C–Pinf^ R)^–M^, (D^+C–Pinf^ R)^–M^, (D^+C+Pinf^ R)^–M^, and (D^–C+Pinf^ R)^–M^. The AMF-colonized plantlets systems without *P. infestans* [i.e., (D^–C–Pinf^ R)^+M^ treatment] were placed between AMF-colonized plantlets systems with *P. infestans* [i.e., (D^–C+Pinf^ R)^+M^ treatment] in the growth chamber set at the conditions described above to investigate the potential involvement of VOCs.

Finally, five additional plantlets from the (D^–C–Pinf^ R)^+M^ treatment were prepared to evaluate root colonization in the receiver plantlets at the start of the experiment (i.e., before infection by *P. infestans*). The complete experiment was conducted twice for 24 and 48 hpi to evaluate repeatability of the results and once at 120 hpi to evaluate a potential late response.

### Plant Growth Parameters

At the end of the experiment, the number of leaves of the receiver plantlets was enumerated and their fresh weight measured prior to immediate storage in liquid nitrogen (see above). Roots were then separated from the gel, their fresh weight measured, and AMF root colonization assessed (see below).

### Cytoplasmic Flow Measurement

Two additional Petri plates of the treatment (D^–C–Pinf^ R)^+M^ were considered to determine the average speed of cytoplasmic flow inside the hyphae. The systems were set up as above and were grown for 8 weeks until a profuse development of hyphae was noticed in the HC. The cytoplasmic flow was assessed in randomly selected runner hyphae developing on the surface of the MSR medium, with a microscope (Olympus BH2, Olympus Optical, GmbH, Germany) at 400X magnifications. The speed was evaluated following the method of Whiteside et al. (2019) by observing the cytoplasmic flow between two graduations of the eyepiece and calculating the time needed for cellular contents to move from one to the other graduation.

### *P. infestans* Assessment on Donor Plant

Leaf infection by *P. infestans* was assessed at 24, 48, and 120 hpi by staining with lactophenol cotton blue. Briefly, at each time point, two inoculated leaves of each infected donor plantlet were cut and 20 μl of lactophenol cotton blue solution added under the cellophane disk. After 24 h, the leaves were cut in 0.5 cm^2^ fragments and placed on slides for observation under a compound microscope (Olympus BH2, Olympus Optical, GmbH, Germany) at 80× magnifications. The observations were compared to an infection scale developed on Bintje placed in the MDP *in vitro* culture system. Three stages were identified: stage 1 (24 hpi) sporangia germination, stage 2 (48 hpi) first hyphae branching and plant tissue infection, and stage 3 (120 hpi), spreading of the hyphae on the leaf surface with the first symptom observed (Supplementary Figure S1).

### Root Colonization by AMF

The roots of the receiver plantlets were cut in 3 cm long pieces, cleared with KOH (10%) at 60°C for 45 min, rinsed with deionized water and then with 1% HCl before staining with a solution of 1% HCl and 2% blue ink (Parker^®^, United States). The roots were subsequently transferred in a solution of lactoglycerol (1v:1v:1v—lactic acid/glycerol/H_2_O) and kept at 4°C. Root colonization was assessed following McGonigle et al. (1990). A mean of 150 intersections was observed under a compound microscope (Olympus BH2, Olympus Optical, GmbH, Germany) at 100X magnifications. Percentages of total root colonization (%RC) and arbuscules (%A) were assessed.

### RNA Extraction

The frozen leaves of each replicate were ground in liquid nitrogen (−196°C). Total RNA extraction was done using the RNeasy Plant Mini Kit (Qiagen, United States) following manufacturer recommendations with a slight modification (i.e., for water elution, a 5 min step of incubation on the column at room temperature was performed instead of 1 min). The total RNA was treated with TURBO DNA-free^TM^ Kit (Thermo Fisher Scientific, United States), according to the manufacturer protocol. Concentration of each sample was measured using NanoDrop^®^-ND 1000 UV–vis Spectrophotometer (NanoDrop Technologies, United States) and RNA purity estimated from the A260/A280 absorbance ratio.

Following total RNA extraction, reverse transcription (RT) of 0.5 mg of total RNA was performed with the Transcriptor High Fidelity cDNA Synthesis Kit (Roche, Montreal, QC, Canada), according to the manufacturer instructions. For each RNA sample, a reaction without RT enzyme was performed as a control for contamination by genomic DNA.

### RT-qPCR

Three reference genes (Table 1) were selected [i.e., Name (abbreviations) and GenBank ID, respectively] using Refinder software^3^. Fifteen target genes involved in plant defense or signaling pathways were selected (Table 1). The qPCR was made using LightCycler FastStart Essential DNA Green Master in 10 μl volume of reaction formed as follow; 5 μl Master mix, 0.5 μl of each primer from the pair, 1.5 μl H_2_O PCR grade, and 2.5 μl DNA. The cycling conditions were: 10 min at 95°C, 40 cycles of denaturation (95°C, 10 s)/annealing (60°C, 10 s)/extension (72°C, 15 s) and finalized by a standard melting curve analysis (95°C). Normalization was achieved for each experiment separately using the geometric means of the reference genes (i.e., *Ubc*, *EF1-*α, *GAPDH*) and the “Pfaffl” method (Pfaffl, 2001). The results were then expressed as means of fold change in target genes expression in each of the following treatments [(D^+C–Pinf^ R)^–M^, (D^+C+Pinf^ R)^–M^, (D^–C+Pinf^ R)^–M^, (D^–C–Pinf^ R)^+M^, (D^+C–Pinf^ R)^+M^, (D^+C+Pinf^ R)^+M^, and (D^–C+Pinf^ R)^+M^] as compared to the (D^–C–Pinf^ R)^–M^ treatment. Two experiments were conducted independently for 24 and 48 hpi and one was prolonged to 120 hpi. Significance values of genes fold change were set at *P*-value < 0.05, with an additional cut off value of 2.5-fold change.

**TABLE 1:** Primers used in the study.

Pathways	Name (abbreviation)	Accession number or reference	Primer sequence 5′-3′ (forward)	Primer sequence 5′-3′ (reverse)
Reference genes	Ubiquitin conjugating enzyme-like (*Ubc*)	DQ241834	TGATGGTTACCCATTTGAGCC	ACTGGTCCTTCAGGATGTC
	Glyceraldehyde-3-phosphate dehydrogenase (*GADPH*)	AF527779	GGACATTGTCTCCAACGC	ATGAGACCCTCCACAATGC
	Elongation factor 1-alpha (*EF1-*α)	AB061263	ATTGGAAACGGATATGCTCCA	TCCTTACCTGAACGCCTGTCA
SA	Mitogen-activated protein kinase (*MAPK*)	AB206552	CCAAGTAACCTCTTGCTAAATGC	CTGTCATATTCTCGTTCTCTAGG
	Pathogenesis related 1 (*PR-1*)	Burra et al., 2015	AACACTCTGGTGGCCCTTAC	AGCACAACCGAGACGTACTG
	Pathogenesis related 2 (*PR-2*)	AJ009932	TATCATCAGCAGGGTTGCAAA	TCGCGAAAAATGCTATTTCTAGG
	Pathogenesis related 3 (*PR-3*)	Büchter et al., 1997	GGCTGCCTTTTTCGGTCAAA	CCTTGTCCAGCTCGTTCGTA
	Pathogenesis related 9 (*PR-9*)	AJ401150	AAGAAACAACACCAGGGCAC	TGCCCTCAAGCTGAAGAAAT
	Gluthatione-S-transferase 1 (*GST1*)	J03679	TTCTAGCCACCAGATTTGACC	ACATATTCCCTATATTTTTGGAGTGAGTA
	Putative regulatory protein Non-expressor of PR genes 1 (*NPR1*)	XM_006357647.2	GGTGCACCGATGCATTTTGT	TCAGCTCCTTGAGTTCCAGC
	WRKY transcription factor 6 gene (*WRKY6*)	LOC102577893	ATCAAAATTCCAAAGACCCTCC	ATGTTATGTCATCTGGGGTTTAC
	Osmotine like (*OSM*)	Ruiz et al., 2005	GAGGTACGCAACACCTGTCCATAC	GCTAGGGTGTTTGGCGATTTAC
JA/ET	Phenylalanine ammonia lyase (*PAL*)	X63104	GGATATGCCATCGAACTTTGAGA	ACAAATAATGGCATGGATGAGG
	Pathogenesis related 1 basic (*PR-1b*)	AJ250136	TGGTGATTTCACGGGGAGGGCA	TCCGCACACTTGTCCGCTTGCA
ET	Ethylene response factor 3 (*ERF3*)	EF091875	CCTGTTAAAAATGAAATCAATCGGAGTCC	CGGCGATGATGAATCAACCATAAC
JA	Lipoxygenase (*LOX*)	Y18548	GAGTTCTCCTCATGGTGTTCGTTTA	AGTAGTCTGACACCCAACTT
	*Allene oxide cyclase* (*AOC*)	AY135641	GCTACCCTCTGCCTTCCAAA	GGAAGCAGTAGTGGAGGTGG
	12-Oxophytodienoate reductase 3 (*OPR3*)	Halim et al., 2009	AATCCACTCAGCCTTGGCTTAGCAG	GTCCATTGCTTCCATTTCCTTGAA

### Statistical Analyses

Data analyses were performed with JMP^§^ Pro statistical software version 14.0.0 (SAS Inc., Canada) with a linear mixed model, where “Treatment” and “Time” were regarded as fixed factors and “Experiment” as random factor to account for potential differences between experiments. In the case of non-normality and/or unequal variances, data were log or ln transformed prior to running the linear mixed model. Differences between means were subsequently tested by Tukey’s test; significant values were set at *P* < 0.05. Analysis of the variability between experiments for each target genes was made, and proportion of variability explain by the factor experiment was never significant, *P* < 0.05 (Supplementary Table S1), therefore results are shown as means of both experiments for 24 and 48 hpi and as means of one experiment for 120 hpi.

## Results

### Arbuscular Mycorrhizal Colonization

Root colonization of the potato plantlets in the HC (i.e., the uninfected receiver plantlets) was assessed prior (i.e., time 0 h) or 24, 48, and 120 hpi by *P. infestans* of the leaves of potato plantlets in the RC (i.e., the infected donor plantlets). This corresponded to 12, 13, 14, and 17 days of contact of the receiver plantlets with the extraradical mycelium network (Table 2).

**TABLE 2:** Percentage of total roots (% RC) and arbuscules (% A) colonization of the potato receiver plantlets—R (i.e., plantlets grown in the HC) connected to the potato donor plantlets—D (i.e., plantlets grown in the RC) via the extraradical mycelium of *R. irregularis* MUCL 41833.

Treatment	%RC			%A		
	24 h	48 h	120 h	24 h	48 h	120 h
(D^–C–Pinf^ R)^+M^	19.0 ± 18.7	31.7 ± 19.1	32.9 ± 30.4	6.7 ± 9.5	11.6 ± 7	13.0 ± 13.4
(D^+C–Pinf^ R)^+M^	18.7 ± 10.4	13.5 ± 7	38.4 ± 30.8	5.2 ± 2.9	4.4 ± 3.3	17.0 ± 15.7
(D^+C+Pinf^ R)^+M^	18.9 ± 10.3	13.7 ± 9.9	29.6 ± 25.9	6.6 ± 4.2	4.3 ± 3.2	8.9 ± 11
(D^–C+Pinf^ R)^+M^	14.7 ± 10	23.2 ± 14.9	13.6 ± 15.3	4.8 ± 3.9	7.3 ± 5.7	4.9 ± 6.7

**Mixed model**			***P*-values**			

Treatment	0.24024			0.18423		
Time	0.08084			0.15094		
Treatment vs Time	0.19568			0.22407		

*The different treatments were donor plantlets colonized or not by the AMF (+M, −M), their shoot infected or not by *P. infestans* (+Pinf, −Pinf), and the medium in the RC cut or not (+C, −C). The measurements were made 24, 48, and 120 h post-infection (hpi) by *P. infestans* of the donor plantlets. Data are means ± standard error of eight replicates at 24 h, 48 h and four replicates at 120 h. Main effects and interactions between the factors “Treatment” and “Time” are presented with significance values set at *P* < 0.05 (mixed model). Data are means ± standard error of eight replicates at 24 h, 48 h and four replicates at 120 h. Main effects and interactions between the factors “Treatment” and “Time” are presented with significance values set at *P* < 0.05 (mixed model).*

At the time of inoculation of *P. infestans* (0 h), the %RC and %A of the receiver plantlets grown in the HC were 19.9 ± 17.2 and 6.3 ± 6.1%, respectively, and did not differ significantly from the values at 24, 48, and 120 hpi by *P. infestans* (*P* > 0.05), whatever the treatment. No significant effect of the factor “Treatment,” “Time,” or interactions between both factors was observed on %RC and %A (Table 2). No traces of AMF colonization were observed in the receiver potato plantlets grown in the non-AMF treatments.

### Plant Growth Parameters

Root fresh weight (RFW) and leaf fresh weight (LFW) as well as number of leaves of the receiver plantlets grown in the HC were measured 24, 48, and 120 hpi by *P. infestans* of the donor plantlets grown in the RC (Table 3). A significant effect of the factor “Time” was noticed on RFW, LFW, and number of leaves of the receiver plantlets grown in the HC. Whatever the treatment a significant increase of the three parameters was noticed at 48 and 120 hpi as compared to 24 hpi by *P. infestans* (*P* < 0.05). Conversely, no effect of the factor “Treatment” or interactions between “Time × Treatment” was observed whatever the time of observation and parameter (*P* > 0.05).

**TABLE 3:** Root fresh weight (RFW), leaf fresh weight (LFW), and number of leaves of the receiver potato plantlets—R (i.e., plantlets grown in the HC) connected to the donor potato plantlets—D (i.e., plantlets grown in the RC) via the extraradical mycelium of *R. irregularis* MUCL 41833.

Treatment	RFW (mg)			LFW (mg)			Number of leaves		
	24 h	48 h	120 h	24 h	48 h	120 h	24h	48 h	120 h
(D^–C–Pinf^ R)^–M^	321.3 ± 198.7	303.8 ± 196.5	380.0 ± 202.1	151.3 ± 63.3	190.0 ± 89.3	211.3 ± 198.7	8.5 ± 3.9	9.1 ± 3.4	9.5 ± 3.8
(D^–C+Pinf^ R)^–M^	280.0 ± 233.7	356.3 ± 226	296.3 ± 131.7	185.0 ± 111.4	205.0 ± 96.4	208.8 ± 233.7	8.5 ± 3.3	9.0 ± 3.8	9.0 ± 3.5
(D^+C–Pinf^ R)^–M^	240.0 ± 175.4	197.5 ± 86.1	280.0 ± 118.7	168.8 ± 87.1	191.3 ± 84.4	172.5 ± 175.4	8.3 ± 3.7	8.5 ± 3.3	8.8 ± 3.3
(D^+C+Pinf^ R)^–M^	190.0 ± 123.2	341.3 ± 92.8	412.5 ± 167.3	175.0 ± 85.5	192.5 ± 59.9	216.3 ± 123.2	8.4 ± 3.1	8.6 ± 2.9	8.9 ± 3.1
(D^–C–Pinf^ R)^+M^	282.5 ± 246.6	327.5 ± 287.4	298.8 ± 51.9	141.3 ± 68.5	190.0 ± 104.9	190.0 ± 246.6	8.0 ± 2.9	9.1 ± 4.4	9.1 ± 3.2
(D^+C–Pinf^ R)^+M^	252.5 ± 159.3	195.0 ± 89	287.5 ± 54.7	164.4 ± 69	161.3 ± 74.1	183.8 ± 159.3	8.1 ± 3	8.4 ± 3.4	8.6 ± 3.8
(D^+C+Pinf^ R)^+M^	203.8 ± 49.8	193. ± 75	285.0 ± 137.4	186.3 ± 46	196.3 ± 32	201.3 ± 49.8	9.0 ± 3.4	8.5 ± 3	9.0 ± 3.5
(D^–C+Pinf^ R)^+M^	341.3 ± 225.9	323.2 ± 172	418.8 ± 160.9	173.8 ± 71.9	205.0 ± 95.5	211.3 ± 225.9	8.1 ± 3.4	9.0 ± 3.6	9.3 ± 4.2

**Mixed model**	***P*-values**								

Treatment	0.2161			0.3219			0.3677		
Time	**0.0001**			**0.0004**			**0.0009**		
Treatment vs Time	0.4080			0.9933			0.8773		

*The different treatments were donor plantlets colonized or not by the AMF (+M, −M), their shoot infected or not by *P. infestans* (+Pinf, −Pinf) and the medium in the RC cut or not (+C, −C). The measurements were made 24, 48, and 120 h post-infection (hpi) by *P. infestans* of the donor plantlets. Data are means ± standard error of eight replicates at 24 h, 48 h and four replicates at 120 h. Main effects and interactions between the factors “Treatment” and “Time” are presented with significance values in bold and set at *P* < 0.05 (mixed model).*

### Cytoplasmic Flow

The speed of cytoplasmic flow was measured on five independent hyphae. The speed varied from 0.75 to 1.38 cm h^–1^ with a mean cytoplasmic flow speed of 0.96 ± 0.25 cm h^–1^.

### Leaf Infection of the Donor Plantlets by *P. infestans*

The successful infection of the donor plantlets by *P. infestans* [i.e., in the treatments: (D^–C+Pinf^ R)^–M^; (D^+C+Pinf^ R)^–M^; (D^+C+Pinf^ R)^+M^; (D^+C+Pinf^ R)^+M^] was evaluated on two leaves per plantlet, using lactophenol cotton blue staining. The infection stage was compared to a determination key developed for *P. infestans* under similar growth conditions (Supplementary Figure S1). Whatever the treatment all the plantlets inoculated by the pathogen were infected and corresponded to the three stages (i.e., 24, 48, or 120 hpi) from the key scale. No impact on the stage of pathogen development of the different treatments was observed.

### Gene Expression Analysis in the Receiver Plants

**FIGURE 2:**
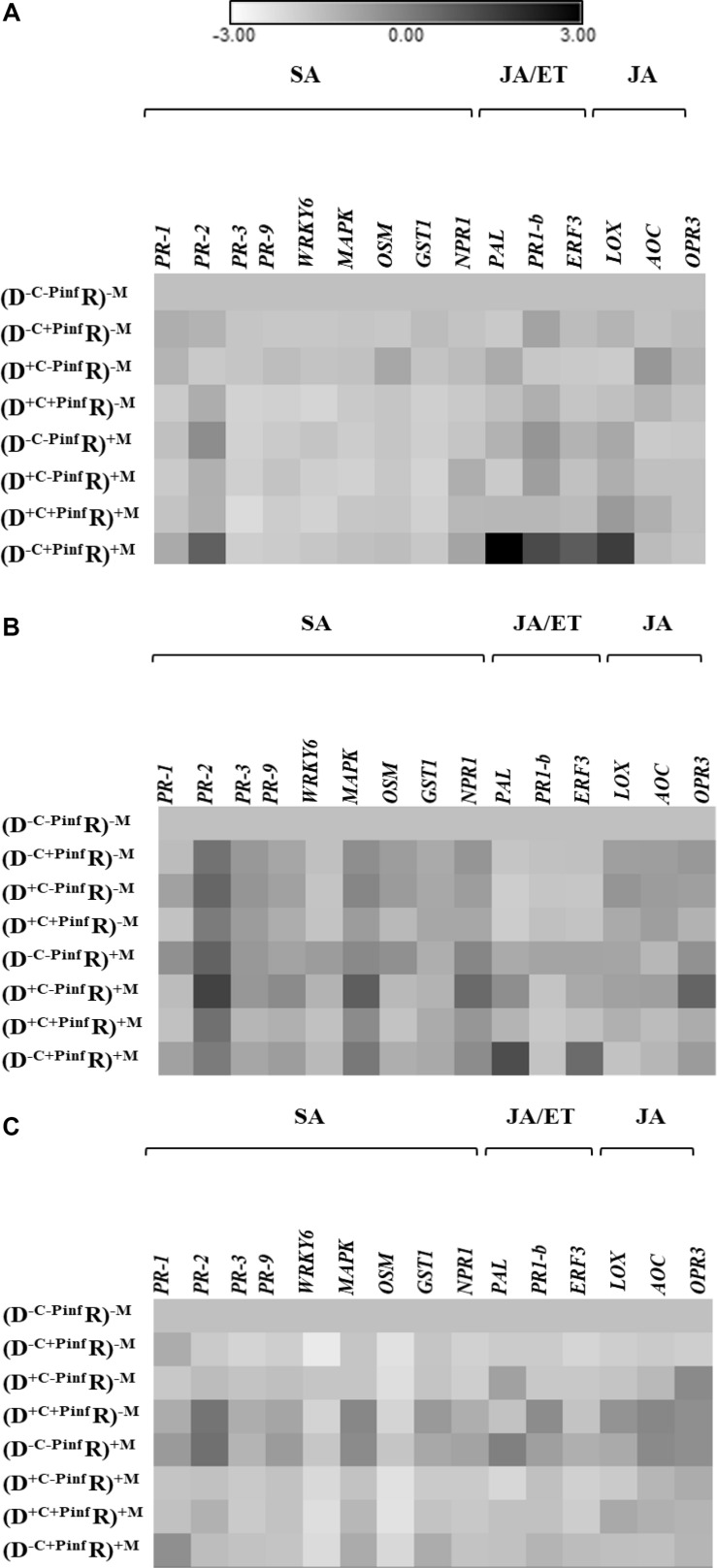
Heat map of the 15 genes relative expression; upregulated and downregulated ratio (in ln) in the leaves of potato receiver plantlets—R (i.e., plantlets grown in the HC), **(A)** 24 h, **(B)** 48 h, and **(C)** 120 h post inoculation of a donor—D plantlet by *P. infestans* (i.e., plantlets grown in the RC). The different treatments were donor plantlets colonized or not by the AMF (+M, −M), their shoot infected or not by *P. infestans* (+Pinf, −Pinf), and the medium in the RC cut or not (+C, −C). Fold change of gene expression of the treatment used for the normalization (D^–C–Pinf^ R)^–M^ is equal to 1. Quantitative real-time RT-PCR was used to detect the transcripts of 15 target genes. Seven salicylic acid (SA) dependent genes; pathogenesis related 1 (*PR-1*), pathogenesis related 2 (*PR-2*), pathogenesis related 3 (*PR-3*), pathogenesis related 9 (*PR9*), WRKY transcription factor 6 gene (*WRKY6*), mitogen-activated protein kinase (*MAPK*), osmotine like (*OSM*), gluthatione-S-transferase 1 (*GST1*), putative regulatory protein non-expressor of PR genes 1 (*NPR1*). Two jasmonic acid (JA) and ethylen (ET) dependent genes; phenylalanine ammonia lyase (*PAL*), pathogenesis related 1 basic (*PR-1b*). One ET dependent gene: ethylene response factor 3 (*ERF3*). Three JA dependent genes: lipoxygenase (*LOX*), allene oxide cyclase (*AOC*), OPDA reductase 3 (*OPR3*). Every square are means of eight replicates at 24 h and 48 hpi and four replicates at 120 hpi (Supplementary Table S1 Expression fold change).

The transcript of 15 target genes (Figure 2) in the leaves of the receiver plantlets in the HC was assessed 24, 48, and 120 hpi by *P. infestans* on the leaves of the donor plantlets in the RC (Figure 1).

At 24 hpi (Figure 2A), four defense genes were significantly upregulated in the (D^–C+Pinf^ R)^+M^ treatment. Indeed, *PR-1b*, *ERF3*, *LOX*, and *PAL* were upregulated by 5.8, 4.8, 9.8, 26.5-fold, respectively. No significant variation of the genes relative expression was observed in the treatments without AMF regardless of *P. infestans* inoculation of the donor plantlets [i.e., in the (D^–C–Pinf^ R)^–M^; (D^+C–Pinf^ R)^–M^; (D^–C+Pinf^ R)^–M^; (D^+C+Pinf^ R)^–M^ treatments]. Similarly, no significant variation of the genes relative expression was induced by the AMF in the (D^–C–Pinf^ R)^+M^; (D^+C–Pinf^ R)^+M^; (D^–C+Pinf^ R)^+M^; (D^+C+Pinf^ R)^+M^ treatments, or by the cut in the RC compartment in the (D^+C–Pinf^ R)^–M^; (D^+C+Pinf^ R)^–M^; (D^+C–Pinf^ R)^+M^; (D^+C+Pinf^ R)^+M^ treatments. In the leaves of the receiver plantlets, no significant modification of gene expression was measured in treatment without an active CMN linking infected donor to receiver plantlets. Furthermore, transcript analysis did not reveal any variation in the relative expression ratio of the target genes regardless of the treatments at 48 or 120 hpi by *P. infestans* (Figures 2B,C).

## Discussion

It is only in the recent decade that the role of CMNs in the transmission of warning signals from infected to uninfected plants has been firmly demonstrated (Song et al., 2010; Babikova et al., 2013a; Gilbert and Johnson, 2017). However, the direct contribution of connecting hyphae can only be ascertained in a context where indirect effects, attributed to shift in microbial communities or biofilm formations or to diffusion of chemical or volatile signals through the substrate, can be excluded. Here, a strict *in vitro* culture system was used to investigate warning signals transmission from donor potato plantlets infected by *P. infestans* to uninfected receiver potato plantlets linked by a CMN of the AMF *R. irregularis*. The plantlets were grown in contiguous compartments, physically separated by a plastic wall, while the AMF crossed the wall and interconnected the plantlets. No significant elicitation of any of the genes considered in this study was noticed in the receiver plantlets at 48 and 120 hpi by *P. infestans* of the donor plantlets, while a significant over-expression of *PR-1b*, *PAL*, and *ERF3* (JA/ET pathway) and *LOX* (JA pathway) transcripts was observed at 24 hpi. This demonstrated the fast and transitory transmission of warning signals from infected to uninfected plantlets via the sole ERM and supported the indubitable role of CMN in providing a route for activating defense responses in uninfected plantlets.

The bi-compartmented experimental system was adequate to investigate signal transmission from infected to uninfected plantlets. Indeed, the CMN was established within 12 days as earlier reported (Gallou et al., 2010) and root colonization of the receiver plantlets reached a mean value close to 20%, at the time of infection by *P. infestans*. Similarly, leave infection of the donor plantlets by *P. infestans* was clearly observed at 24, 48, and 120 hpi by the pathogen with sporangia germination, first hyphae branching and plant tissue infection, and spreading of the hyphae on the leaf surface with the first symptom observed, respectively. Within the experiment time frame, neither *P. infestans* nor the cutting of the medium in the RC impacted root colonization, as earlier observed Gallou et al. (2011), or plant growth parameters of the received plantlets. Only the factor “time” induced an increase in plant growth parameters with significant higher values at 48 and 120 hpi as compared to 24 hpi, suggesting an actively development of the plantlets.

In our *in vitro* cultivation system, microbial communities (and thus biofilms) were absent. Signal transmission from diseased to healthy plant was thus exclusively related to the hyphal network connecting both plants. This does not exclude that biofilms may play a role in signal transmission but prove at least that hyphae in absence of any interaction with microbial communities are able to do so. Indeed, bacterial biofilms have been hypothesized as a potential signal carrier (Barto et al., 2012) and shift in communities has been associated to AMF (Marschner et al., 2001). Here, nor biofilm formation neither contamination of the growth medium was noticed. Diffusion of chemical compounds (Gilbert and Johnson, 2017) via the growth medium could also be discarded in our system because both compartments were physically separated by a plastic wall with only hyphae connecting the plantlets. However, it is not excluded that signals can be transmitted by capillarity along the hyphae, which is another direct pathway along the hyphae (Barto et al., 2012). Furthermore, the root systems of the donor and receiver plantlets were potentially exposed to the same VOCs accumulated inside the Petri plates. To investigate this pathway, a piece of medium was removed along the RC in the AMF or non-AMF treatments with and without *P. infestans*. Similarly, the potential involvement of VOCs released by the aerial parts of the plantlets was controlled by placing AMF-colonized plantlets systems without *P. infestans* [i.e., (D^–C–Pinf^ R)^+M^ treatment] between AMF-colonized plantlets systems with *P. infestans* [i.e., (D^–C+Pinf^ R)^+M^ treatment]. In both cases, no response was measured in the receiver plantlets, indicating that VOCs were most likely not involved in the signaling process as earlier suggested (Song et al., 2010). From these observations, it appears that the most plausible mechanism for the transmission of warning signals is by chemicals along or with more certainty inside the hyphae.

Plant defense genes activation was observed 24 hpi by *P. infestans*, while no effect was noticed 48 and 120 hpi. Earlier studies have reported response in uninfected plants already 18 hpi by *Alternaria solani* (Song et al., 2010) or even 6 hpi by *Spodoptera litura* (Song et al., 2014) and wounding signal transmission from leaves to roots via the JA pathway has been shown within 90 min (Zhang and Baldwin, 1997). Mitochondria, nucleus, and fat droplets speed movement in hyphae was evaluated at 15 cm day^–1^ (Giovannetti et al., 1999) but cytoplasmic flow was calculated at 12.6 cm h^–1^ in pot conditions (Cox et al., 1980). In a recent study conducted *in vitro*, the cytoplasmic/protoplasmic flow was estimated between 0.7 and 18 cm h^–1^ (Whiteside et al., 2019) and in our experiment it was estimated around 0.96 cm h^–1^. Therefore, movement of cytoplasmic/protoplasmic material between hyphae situated at the longest distance of a Petri plate (i.e., 90 mm) is expected to occur within an approximate of 10 h, to which should be added the time needed from a signal to be transmitted from infected shoot of the donor plantlets to hyphae in the RC and from hyphae in the HC to the uninfected shoot of the receiver plantlets. This should take less than 24 h and allow elicitation of defense mechanisms in the healthy receiver plants (Johnson and Gilbert, 2015; Gilbert and Johnson, 2017).

The highest response in the receiver plantlets was generally recorded between 48 and 100 hpi of the pathogen and decreased thereafter (Gilbert and Johnson, 2017), while in our experiment it was within 24 hpi. However, the current experiment was conducted in 90 mm Petri plate as compared with pot experiments where plant were separated by a distance of 15 cm (Song et al., 2010) or 20 cm (Babikova et al., 2013b). The smaller distance between roots in our system may have shortened the time necessary for defense activation and may have contributed to decrease the time required before defense response return to basal level. Indeed, even if the duration over which the signals are effective is still unknown (Gilbert and Johnson, 2017), the receiver plant response seems to be transient. If the receiver plant is not challenged by a pathogen/attacker, then the gene expression returns to basal level and the short distance between plantlets in the *in vitro* system may explain the absence of gene expression 48 and 100 hpi. Babikova et al. (2014) have evaluated the speed of electrical signal such as calcium signal at 40 m s^–1^, therefore short distances between plants (i.e., around 10 cm) should not have impacted the time for gene defense elicitation in the receiver plantlets. Therefore, it could be hypothesized, but still should be confirmed experimentally, that electric signal is not involved (Johnson and Gilbert, 2015).

The first report of warning signals transmission between plants via CMN was made by Song et al. (2010). These authors demonstrated the activation of plant defense genes of both SA and JA pathways (*PR-1*, *PR-2*, *PR-3*, *PAL*, *LOX*, *AOC*). In another study, Song et al. (2014) used tomato mutants defective in JA biosynthesis and observed that they were unable to induce defense response, strongly suggesting that the JA signaling pathway was required. Our results seem to indicate similar results. Indeed, JA dependent gene was upregulated as well as *PR-1b*, *PAL*, and *ERF3* (JA/ET pathway). Furthermore, no activation of SA dependent genes was measured in our study.

Our results suggested a priming phase (Mauch-Mani et al., 2017) of plant defense with upregulation of four genes (i.e., *PR-1b, PAL*, *ERF3*, *LOX*) in the (D^–C+Pinf^ R)^+M^ treatment. The priming phase refers to the biological process of acquiring priming, which takes place from the initial stimulation through the exposure to a challenging stress (Mauch-Mani et al., 2017). Priming demonstration would have required subsequent infection of the receiver plant by *P. infestans*, but was not the aim of this experiment. However, priming phase involves notably signalization cascade activation (Balmer et al., 2015; Conrath et al., 2015). *ERF3* has been shown to play a role in the activation of plant systemic defense system in potato plants (Velivelli et al., 2015) and against leaf infection by *P. infestans* (Gallou et al., 2011). The basic *PR-1b* protein (i.e., JA/ET dependent) is involved in innate immunity resistance of potato against *P. infestans* (Vleeshouwers et al., 2000) and was reported in AMF-colonized potato plants challenged by *P. infestans* (Gallou et al., 2011). Priming of potato plants also induced the activation of *LOX* which is involved in systemic signaling (Halim et al., 2009). *PAL* was also used as a JA/ET dependent gene involved in innate immunity against pathogens (Song et al., 2010) and was shown in AMF-colonized potato plants challenged by *P. infestans* (Gallou et al., 2011). The upregulation of these four genes (*PR-1b*, *PAL*, *ERF3*, and *LOX*) clearly suggested the elicitation of the ET/JA dependent defense gene in uninfected potato plants connected to infected potato plants.

## Conclusion

We have clearly demonstrated the transitory induction of JA/ET (i.e., *PAL*, *PR-1b*, and *ERF3*) and JA (*LOX*) dependent genes in uninfected receiver plantlets connected via a CMN to a diseased plantlet. These findings reveal the direct involvement of hyphae in the transmission of the warning signals and their indubitable role in providing a route for activating defense responses in uninfected plants. The results further raise crucial questions about the specificity of signal transfer and nature of the signaling processes involved.

## Data Availability Statement

The datasets analyzed during the current study are available from the corresponding author on reasonable request.

## Author Contributions

P-LA, SC, and SD conceived the experiments and reviewed the manuscript. P-LA and FN conducted the experiments. P-LA analyzed the results and wrote the manuscript.

## Conflict of Interest

The authors declare that the research was conducted in the absence of any commercial or financial relationships that could be construed as a potential conflict of interest.

## Footnotes

1 http://www.mycorrhiza.be/ginco-bel

2 http://bccm.belspo.be/about-us/bccm-mucl

3 http://150.216.56.64/referencegene.php?type=reference
