# Supplementary material for: Common Mycorrhizal Network Induced JA/ET Genes Expression in Healthy Potato Plants Connected to Potato Plants Infected by Phytophthora infestans
Source: Front Plant Sci. 2020 May 25;11:602. doi: 10.3389/fpls.2020.00602 (PMC7261899; doi:10.3389/fpls.2020.00602)
Supplement: Supplementary file 1 [file Image_1.pdf]

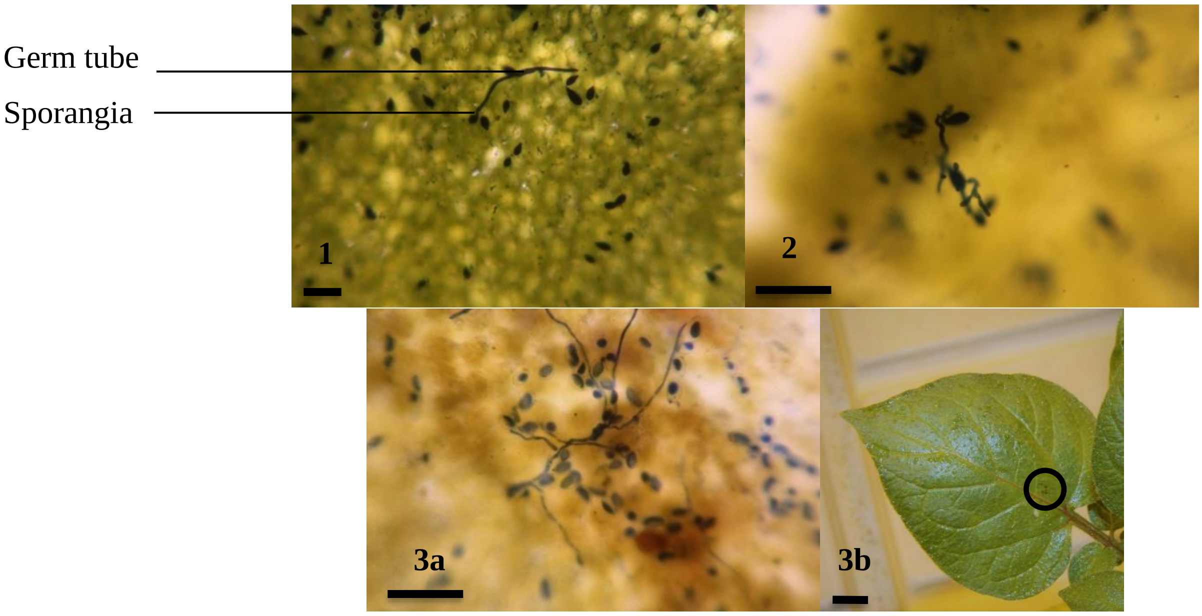

Supplementary Figure S1. Early stages of *P. infestans* infection on potato plants grown *in vitro*. (1) germinating sporangia 24 h post inoculation. (2) Hyphae branching and plant tissue infection 48 h post inoculation (3a) spreading of hyphae on the leaf surface with the first symptom observed 120 h post inoculation. (3b) Round circle showing first necrosis. Scale bar = 0.25mm for Picture 1, 2, 3a and scale bar = 5mm for 3b.
